# Long-term effects of siponimod on cardiovascular and autonomic nervous system in secondary progressive multiple sclerosis

**DOI:** 10.3389/fphar.2024.1431380

**Published:** 2024-09-19

**Authors:** Victor Constantinescu, Rocco Haase, Katja Akgün, Tjalf Ziemssen

**Affiliations:** ^1^ Center of Clinical Neuroscience, Department of Neurology, University Hospital Carl Gustav Carus, Dresden, Technical University of Dresden, Dresden, Germany; ^2^ Department of Neurology, University of Medicine and Pharmacy “Grigore T. Popa” Iasi, Iasi, Romania

**Keywords:** multiple sclerosis, siponimod, autonomic nervous system modulation, cardiovascular effect, baroreflex sensitivity

## Abstract

**Background:**

Siponimod, a second-generation, selective sphingosine 1-phosphate receptor (S1PR) 1 and 5 modulator, represents an important therapeutic choice for active secondary progressive multiple sclerosis (SPMS). Besides the beneficial immunomodulatory effects, siponimod impacts cardiovascular function through S1PR1 modulation. Short-term vagomimetic effects on cardiac activity have proved to be mitigated by dose titration. However, long-term consequences are less known.

**Objectives:**

This study aimed to investigate the long-term impact of siponimod on cardiac autonomic modulation in people with SPMS (pwSPMS).

**Methods:**

Heart rate variability (HRV) and vascular hemodynamic parameters were evaluated using Multiple Trigonometric Regressive Spectral analysis in 47 pwSPMS before siponimod therapy and after one, three, six and 12 months of treatment. Autonomic activation tests (tilt test for the sympathetic and deep breathing test for the parasympathetic cardiac modulation) were performed at each examination.

**Results:**

pwSPMS preserved regular cardiovascular modulation responses during the autonomic tests reflected in the variation of several HRV parameters, such as RMSSD, pNN50, total power of HRV, high-frequency and low-frequency bands of the spectral domain or hemodynamic vascular parameters (Cwk, Zao, TPR, MAP) and baroreflex sensitivity (BRS). In the long-term follow-up, RMSSD, pNN50, total power, BRS and CwK presented a significant decrease, underlining a reduction of the parasympathetic and a shift towards sympathetic predominance in cardiac autonomic modulation that tends to stabilise after 1 year of treatment.

**Conclusion:**

Due to dose titration, the short-term effects of siponimod on cardiac autonomic modulation are mitigated. The long-term impact on cardiac autonomic modulation is similar to fingolimod. The autonomic activation tests showed normal cardiovascular responses during 1-year follow-up in pwSPMS, confirming the safety profile of siponimod. Further research on autonomic function could reveal whether the observed sympathetic activation is a compensatory response to S1P signaling intervention or a feature of the disease, while also shedding light on the role of S1PR modulation in MS.

## 1 Introduction

Sphingosine 1-phosphate (S1P) signaling pathway is involved in a broad spectrum of physiological and pathophysiological processes, primarily through the activation of five extracellular S1P receptors subtypes (S1PR1–S1PR5) (McGinley and Cohen, 2021). Modulation on S1PR is a potential target for treatment of various autoimmune disorders (Constantinescu et al., 2022a).

In 2010, fingolimod, an S1PR modulator, was the first oral therapy to be approved for the treatment of the relapsing forms of multiple sclerosis (MS). Since 2019, three second-generation S1PR modulators have been approved: siponimod for relapsing and secondary progressive MS (SPMS) as well as ozanimod and ponesimod for relapsing MS (Roy et al., 2021; Kappos et al., 2021)^.^ These S1PR modulators differ from the specificity for the individual S1PR subtypes (fingolimod: S1PR1, S1PR3-S1PR5; siponimod and ozanimod: S1PR1, S1PR5; ponesimod: S1PR1) which lead among others to different immunological phenotypes (Proschmann et al., 2023).

In MS, the primary clinical efficacy of S1PR modulators is achieved through their coupling with subtype S1PR1, thus regulating immune cell trafficking through sequestration of autoreactive lymphocytes in the lymph nodes and presumably reducing migration and subsequent infiltration into the central nervous system (CNS) (Roy et al., 2021; Sehr et al., 2019; Fischer et al., 2021). Besides the immunomodulatory beneficial outcome in MS, S1P signaling regulates the cardiovascular function via S1PR1–S1PR3 subtypes, which reside on cardiac myocytes, endothelial, and vascular smooth muscle cells (Constantinescu et al., 2022b). This allows the mediation of the peripheral vascular tone. Further, it has been proposed that hypertension pathogenesis may involve S1P downstream signaling activation (Jozefczuk et al., 2020).

S1PR1 plays a prominent role in the regulation of the heart rate (HR) (Mazurais et al., 2002; Forrest et al., 2004) and is responsible for temporary bradycardia and, less commonly, for a delay in atrioventricular (AV) conduction after the initiation of S1PR modulators therapy. These parasympathomimetic effects are transitory and require cardiac monitoring in certain cases. The particular changes reveal that S1PR modulators act initially as agonists, but play an antagonist role after downregulation of S1PR at the cell surface (Camm et al., 2014). Furthermore, HR changes are accompanied by a transient reduction in blood pressure (BP) after the first dose of S1PR modulators. These short-term effects were first described with fingolimod initiation (Li et al., 2017). Consequently, starting with siponimod, a titration dose following the Fibonacci sequence has been applied to induce the internalization (and downregulation) of S1PR at low doses, which are known to have a mild cardiac effect (Legangneux et al., 2013).

Heart rate variability (HRV) represents a simple and non-invasive method for assessing the sympathovagal balance (Li et al., 2017), outlining the cardiac ability to adapt to hemodynamic and pathological conditions. Sympathetic hyperactivation and reduced cardiac vagal modulation associated with low HRV determines higher risk of cardiac arrhythmia and cardiovascular complications. Analyzing HRV alongside hemodynamic parameters such as baroreflex sensitivity (BRS), arterial pressure, and arterial wall properties (such as arterial compliance or peripheral resistance) can help understand the interaction between the autonomic nervous system and cardiovascular function.

People with MS (pwMS) typically experience autonomic nervous system (ANS) dysfunction, and those with SPMS (pwSPMS) are more likely to experience ANS abnormalities than those with relapsing MS (Habek et al., 2022; Merkelbach et al., 2006). Cardiac autonomic function can be assessed using various autonomic tests such as the deep breathing or tilt test. These tests evaluate vagal or sympathetic activation, respectively.

Data on long-term follow-up of the S1PR modulators’ treatment on cardiac autonomic regulation are scarce. Fingolimod treatment over 6 months determined a decrease of parasympathetic cardiac modulation and consistent reduction in the BRS (Racca et al., 2016). Moreover, a slight increase in BP values was mentioned after fingolimod treatment on long-term follow-up. Similar to fingolimod, siponimod also caused a change towards parasympathetic predominance within the first three hours of administration (Habek et al., 2022). However, following 6 months of continued treatment, a shift towards sympathetic predominance was reported (Habek et al., 2022). Given that bradycardia and arterial hypertension, two well-documented side effects of S1PR modulators, are intimately related to parasympathetic and sympathetic activity, this finding is particularly pertinent to the setting of MS therapy with S1PR modulators. As the cardiac autonomic dysregulation is most likely caused by impaired central modulation of sympathetic and parasympathetic outflow (Mahovic and Lakusic, 2007), a dysfunction in the autonomic nervous system may contribute to long-term disability in pwMS (Winder et al., 2019) interdependently. Cardiovascular deconditioning caused by MS-related disability and overall plaque burden may explain the interference with the autonomic activity (Racosta et al., 2015). The length and severity of the disability in pwSPMS might impact the magnitude of the cardiac autonomic response after the S1PR modulator initiation, but there is still insufficient data. The long-term effects of siponimod on ANS function in pwSPMS is less studied, but of great importance as pwSPMS tend to be older and more often present with comorbidities (Ziemssen et al., 2022).

## 2 Aim

As shown for other S1PR modulators (e.g., fingolimod), short- and long-term changes in cardiac autonomic activity are present in people with relapsing–remitting MS (RRMS), we expect that siponimod will impact the sympathovagal cardiac autonomic balance. A similar observation was noted in a six-month study on pwSPMS. As our analysis is based on a 12-month follow-up of pwSPMS treated with siponimod, the primary outcome was to investigate whether continuous administration of siponimod in the long term will cause changes in HRV, BRS and vascular parameters, with a shift towards sympathetic predominance. Our dataset expanded as we studied the time and frequency domain parameters of HRV, along with vascular hemodynamic parameters, in four different scenarios: resting state, deep breathing, tilt test, and return to clinostatism (re-tilt). Our goal was to understand if these autonomic challenges provoke appropriate cardiovascular responses, such as increased parasympathetic activation after deep breathing and re-tilt, and increased sympathetic activation after the tilt test.

## 3 Methods

### 3.1 Patients

Our study included pwSPMS in whom treatment with siponimod was ongoing for at least 1 year. The study was performed at the MS Center, University Hospital “Carl Gustav Carus” Dresden, Germany from February 2020 to June 2022. The diagnosis of SPMS was based on Lublin criteria (Lublin et al., 2014). Before starting treatment with siponimod, pwMS underwent genotype testing for CYP2C9. The results showed that there were no CYP2C9*2*3 or *1*3 genotypes, nor any homozygous genotypes for CYP2C9*3. Therefore, all patients received a maintenance dose of 2 mg of siponimod after titration, according to the label set by the European Medicines Agency (European Medicines Agency, 2020). PwSPMS had highly disease activity despite an adequate treatment with at least one disease modifying therapy. Only pwMS older than 18 years were enrolled. Exclusion criteria included: pwMS who, in the previous 6 months, had a myocardial infarction, unstable angina pectoris, stroke, decompensated heart failure requiring inpatient treatment, or New York Heart Association (NYHA) class III/IV heart failure, or history of second-degree Mobitz type II or, third-degree atrioventricular block, sino-atrial heart block or sick sinus syndrome (in the absence of pacemaker). The pwSPMS were observed during regular clinical visits for 1 year of siponimod treatment: before starting the siponimod treatment, noted as month 0 (M0), after 1 month of treatment (M1), after 3 months of treatment (M3), after 6 months of treatment (M6) and after 12 months of treatment (M12) completing cardiac autonomic battery tests. Presence of medication with known interference on the ANS function (anticholinergics, antihypertensives - beta blockers and diuretics, antiarrhythmics, sympathomimetics, parasympathomimetics), were taken into consideration and pwSPMS meeting this conditions were excluded from the study. Our study initially included 57 pwSPMS, but only 47 remained in the study after applying the exclusion criteria. These patients underwent treatment with siponimod for 1 year. Clinical information including the Expanded Disability Status Scale (EDSS) scores of the patients was collected.

The study was in accordance with relevant guidelines and regulations, and approved by the Institutional Review Board of University Hospital Carl Gustav Carus. This study was carried out according to the Declaration of Helsinki. All the subjects gave written informed consent prior to participation.

### 3.2 Cardiac autonomic tests

ANS testing was performed as part of neurological routine testing for S1PR modulators’ treated MS patients at several time points, at visits M0, M1, M3, M6 and M12. We applied a standardized protocol including resting state and subsequent autonomic activation tests (tilt test, followed by a supine resting period, named re-tilt, and deep breathing test), each test involving a 3-min recording of the physiological parameters (e.g., HR, BP). There was a HR stabilization before starting the evaluation (Ziemssen and Siepmann, 2019). All the tests were performed at the same time range in the morning (10–11 a.m.), after 30 min of resting position in clinostatism, at a constant temperature of 22°C, in a quiet room, in the absence of sounds or human voices, without prior physical effort or ingestion of caffeinated or alcoholic beverages 24 h before the evaluation. All patients were asked to empty the bladder before starting the evaluation. Moreover, the tests were performed at least three hours after breakfast in order to avoid gastric distension.

Data processing was done using MTRS software version 7.3.2.0 (University Hospital, Center for Clinical Neuroscience, Dresden, Germany). Before each analysis, a manual data correction of the recorded ECG artefacts was carried out. The dynamic assessment of HRV by MTRS (Multiple Trigonometric Regressive Spectral) analysis allows a precise evaluation of cardiovascular modulation under different conditions (Li et al., 2018). The evaluation of these function tests has been based on absolute or relative changes in the physiological parameters, as the HR and the BP. The addition of spectral and baroreflex analysis, estimating the BRS, allowed a more detailed interpretation of these tests (Friedrich et al., 2010). This software assesses the HRV time domain and frequency domain parameters, based on the trigonometric regressive spectral analysis (Li et al., 2018). The algorithm of this analysis provides a pure physiological spectrum using only statistical methods (trigonometric regression). Spectral analysis of HR may identify autonomic inputs to the heart (Papaioannou, 2007). The two major spectral bands are illustrative: the high frequency (HF) power of HR (spectral band between 0.15 and 0.4 Hz), which is related to respiratory sinus arrhythmia and, therefore, to parasympathetic cardiovagal tone, and the low frequency (LF) power of HR (spectral band between 0.04 and 0.15 Hz), that reflects the baroreflex modulation of autonomic outflows. Very low frequency band (VLF) power of HR is related to thermoregulatory mechanisms and the activity of the renin-angiotensin system (Heart rate variability, 1996). The LF/HF ratio allows the quantification of the relation between the two branches of the autonomic nervous system (Friedrich et al., 2010). The short-term HRV analysis involved the frequency domain components, such as relative loads of HF, LF and VLF, and the time domain parameters, such as root mean square of successive differences (RMSSD) and percentage of adjacent NN intervals that differ from each other by more than 50 milliseconds (ms) (pNN50) (Shaffer and Ginsberg, 2017). These two time domain parameters reflect the parasympathetic influence on the cardiac rhythm. An insight into the functioning of the autonomic nervous system is gained by analyzing the variation of the R-R interval (RRI). The RRI represents the time between two successive R-waves of the QRS signal on the electrocardiogram, and is affected both by intrinsic properties of the sinus node and by autonomic influences. The RRI and the HR are in a reciprocal relationship. HRV analysis was performed for each visit (M0, M1, M3, M6 and M12) according to the autonomic nervous system testing protocol.

We also used Finometer Pro (Finapres Medical Systems, the Netherlands) to monitor BP non-invasively during autonomic tests with a volume clamp placed on the finger. Transmural pressure changes are monitored and assessed by a computer algorithm. The Finapres automatically follows the changes in the size of the arterial vessels caused by shifts in the smooth muscle tone and generates a calibrated pressure waveform, which is analysed by a pattern recognition programme to provide systolic BP (SBP) and diastolic BP (DBP). Beat-to-beat BP can be studied over a chosen period, and additional hemodynamic parameters can be calculated, such as - reconstructed mean arterial pressure (MAP), total peripheral resistance (TPR), aortic impedance (ZaO) or Windkessel compliance (Cwk). MAP is the average arterial pressure throughout one cardiac cycle, systole, and diastole. A usual formula used to estimate the MAP is DBP +1/3(SBP–DBP) (Berlin and Bakker, 2015). MAP is influenced by cardiac output (CO) and systemic vascular resistance, each controlled by several variables (Berlin and Bakker, 2015). Arterial impedance measures how the arterial system resists and manages blood flow. Arterial compliance specifically describes how elastic and adaptable the arteries are to pressure changes and helps reduce the fluctuations caused by the pulsatile nature. Reduced compliance leads to higher pulsatility and increased systolic pressure, which can strain the cardiac function.

### 3.3 Statistical analysis

Normal distribution of data was visually assessed using quantile–quantile plots. Quantitative characteristics were presented as mean values, followed by standard deviation (SD) or range. Generalized linear mixed models analyses were performed. Model results are presented as mean value with 95% confidence interval (CI). Model estimates below zero were set to zero. Age, gender, body mass index, EDSS, premedication, disease duration, time point of evaluation (M0-M12) and situation (resting state, tilt, re-tilt or deep breathing) and interaction of time point and situation served as fixed factors. *p*-values less than 0.05 were considered as statistically significant. For pairwise comparisons, contrast tests with the Sidak correction were applied. Statistical analyses were performed using the IBM SPSS software (version 28.0, IBM Corporation, Armonk, NY, United States).

## 4 Results

In our study, 31 women and 16 men with an age of 56.74 ± 8.50 years (mean ± SD) provided data for our analyses. The average disease duration was 16.49 ± 9.39 years (mean ± SD) and median EDSS was 6.0 (min: 2.0; max: 7.0). After splitting into two groups, 53.2% of the participants presented with an EDSS score between 6.0 and 7.0, while 46.8% presented with an EDSS between 2.0 and 5.5. The mean BMI (24.90 ± 3.73) was still within healthy range.

In our cohort, heart rate values showed normal dynamics during autonomic challenge tests, increasing after sympathetic activation (tilt test, *p* < .001) and decreasing after parasympathetic activation (deep breathing, *p* < .001). These normal variations were consistent across all time-point evaluations (*p* = .719, Table 1). As expected, RRI values followed the opposite trend by decreasing after the tilt test (*p* < 0.001), corresponding to a physiological sympathetic activation, recovering to previous values after coming back to clinostatism (re-tilt, *p* < .001) and increased after a deep-breathing test (*p* < 0.001).

**TABLE 1:** Heart rate during autonomic tests over the course of 12 months (N = 47).

Examination	Resting state	Tilt test	Re-tilt	Deep breathing test
Month 0	68.48 [65.40; 71.56]	78.14 [73.97; 82.32]	69.00 [65.81; 72.199	66.89 [63.05; 70.74]
Month 1	68.80 [65.47; 72.13]	76.19 [72.11; 80.27]	68.88 [66.00; 71.76]	66.82 [63.55; 70.08]
Month 3	68.18 [65.34; 71.02]	75.31 [71.41; 79.21]	68.28 [65.10; 71.46]	65.72 [62.45; 68.99]
Month 6	68.51 [65.53; 71.49]	76.34 [72.59; 80.09]	69.02 [66.08; 71.95]	66.89 [63.30; 70.48]
Month 12	67.48 [63.91; 71.06]	74.19 [69.98; 78.39]	67.12 [64.20; 70.03]	65.27 [62.41; 68.14]

Estimated marginal means and 95% confidence intervals are displayed. Heart rate values are expressed as beats per minute.

**FIGURE 1:**
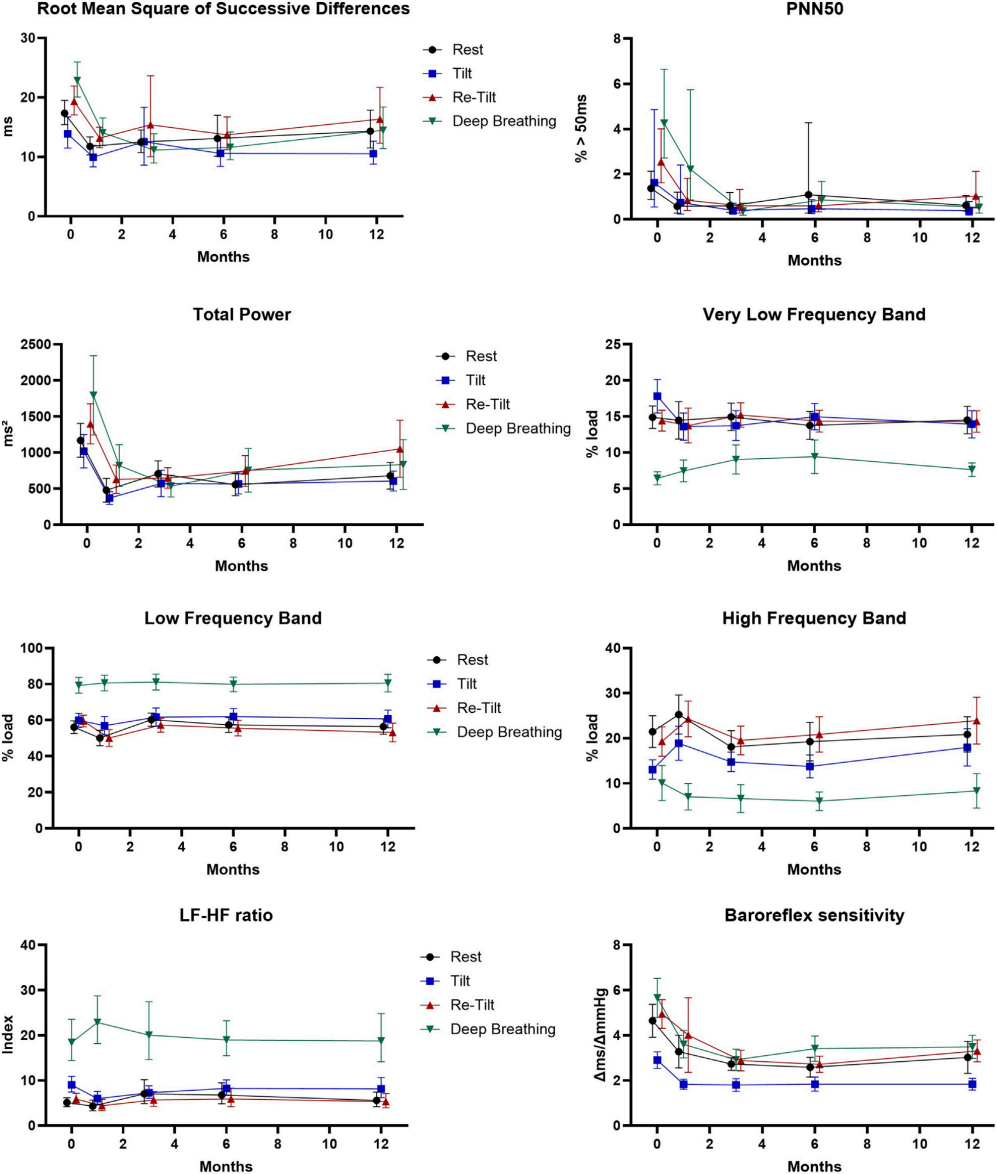
Cardiovascular parameters during the autonomic tests over the course of 12 months (N = 47). Estimated marginal means and 95% confidence intervals are displayed. PNN50: proportion of inter-beat intervals above 50 ms.

The RMSSD parameter illustrates the beat-to-beat variance in HR and is the primary time domain measure used to estimate the vagally mediated changes reflected in HRV (Shaffer et al., 2014). We observed a normal variation of the RMSSD parameter during autonomic tests (Figure 1), with a significant decrease during the tilt test, corresponding to the reduction of vagal modulation (*p* = .001). Regarding the long-term variation, there is a significant decrease (*p* < .001) in the RMSSD values after 1 month of siponimod treatment, maintained for up to 6 months, corresponding to a sympathetic activation that diminishes toward month 12. The reduction in RMSSD values after the first month and the tendency of recovery after 1-year follow-up is present in all four situations proportionally (resting state, tilt, re-tilt and deep breathing) (Figure 1).

Another parameter reflecting the vagal modulation, pNN50, displayed normal variation of its values during autonomic tests, with decreased values after sympathetic activation tests (tilt) and increased values after re-tilt and deep breathing tests (Figure 1). This response was observed at baseline and after 1 month but diminished after 3 months (*p* = .015).

We observed that after 1 month of follow-up, pwMS under siponimod presented significant reduced pNN50 values (*p* < .001), maintained for up to 1 year, compared to M0.

Our results show that overall autonomic activity measured as total power varies according to the autonomic test during all follow-up time points (Figure 1). A persistent and significant decrease (*p* < .001) of the total power of HRV starts with M1 to M12. Regarding spectral analysis, we found a significantly higher relative power of the LF band (LF_rel) and LF/HF ratios of RR intervals and a significantly lower relative power of the HF band (HF_rel) of RR intervals during the tilt phase and the deep breathing test compared to the resting situation (*p* < .001, respectively) maintained during the 12-month follow-up (Figure 1).

In the course of the autonomic tests, we observed normal responses of the BRS, with a decrease consecutive to the tilt test and an increase after the deep breathing test (Figure 1). In the meantime, we noticed a significant reduction in the BRS starting with M1 of treatment with siponimod (*p* < .001), which continued until M6, and a stabilisation at M12.

We calculated the mean MAP during the autonomic tests, and we noticed an increase in the values during the sympathetic activation test (tilt and re-tilt) and a decrease after the deep breathing test, illustrating a regular cardiac autonomic modulation during the challenge (*p* < .001) (Table 2).

**TABLE 2:** Mean arterial pressure during autonomic tests over the course of 12 months (N = 47).

Examination	Resting state	Tilt test	Re-tilt	Deep breathing test
Month 0	101.74 [97.56; 105.91]	105.35 [101.37; 109.33]	105.75 [101.82; 109.67]	99.16 [95.26; 103.06]
Month 1	102.69 [97.87; 107.51]	104.38 [100.24; 108:51]	106.34 [102.09; 110.60]	100.34 [95.84; 104.84]
Month 3	103.50 [98.21; 108.78]	105.55 [99.76; 109.34]	106.48 [101.28; 111.67]	102.18 [96.60; 107.76]
Month 6	100.51 [96.07; 104.96]	101.33 [97.11; 105.54]	103.62 [99.73; 107.52]	96.85 [92.79; 100.92]
Month 12	107.81 [102.64; 112.99]	108.48 [103.51; 113.46]	109.65 [104.97; 114.32]	102.85 [98.44; 107.27]

Estimated marginal means and 95% confidence intervals are displayed. Arterial pressure values are expressed as millimetres of mercury (mmHg).

**FIGURE 2:**
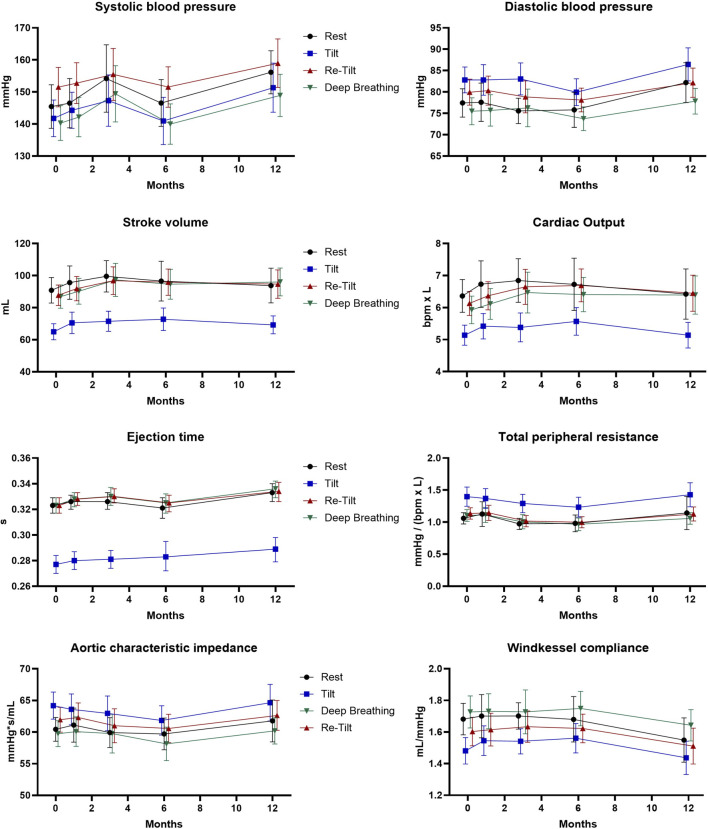
Extended hemodynamic assessment during the autonomic tests over the course of 12 months (N = 47). Estimated marginal means and 95% confidence intervals are displayed.

Other parameters describing the hemodynamics of the arterial system in terms of resistance and compliance are TPR and Cwk, respectively. During the autonomic tests we noted normal vascular responses in pwMS under siponimod treatment (Figure 2), with an increase in mean values of TPR and ZaO after sympathetic activation, underlying a pressor response to adrenergic stimulation, and a decrease in arterial compliance (Cwk).

Age impacts cardiac autonomic modulation. In our cohort, there was a negative correlation between age and RMSSD, pNN50, total power, BRS, DBP, CO and CwK. At the same time, we observed a positive correlation of age with VLF load, TPR, and ZaO. After applying a split for disability in our cohort, participants with a lower EDSS (below 6.0) had a higher total power (+187.9 m^2^), LF-HF ratio (+1.72), VLF load (+2.51%), LF load (+4.36%), EJT (+0.012s) and TPR (+0.136mmHg/bpm*L) but also a lower HF load (−2.99%), cardiac output (−0.63bpm*L) and heart rate (−3.66bpm) across all time points and tests (all *p* < .01).

## 5 Discussion

S1P–S1PRs coupling influences the heart rate, blood pressure, and cardiac autonomic regulation. Animal models with sphingosine kinase knockout mice helped to observe how specific receptor subtypes contribute to cardiac function and completed observations from human studies. The S1P signalling pathways involve the G protein-gated inwardly rectifying potassium channels (GIRK), that are activated after the initial binding of S1PR modulator to S1PR1 (and S1PR3 for fingolimod) on cardiac myocytes (Constantinescu et al., 2022b; Means and Brown, 2009). The atrial GIRK channels, often attributed to the atrial muscarinic–gated potassium channels (IKACh), interfere with the cholinergic agonists’ modulation of action potential duration and the excitability of ventricular myocytes (Lee et al., 2018; Posokhova et al., 2010; Liang et al., 2014), triggering a vagal response, such as bradycardia and delay in atrioventricular conduction. Simultaneously, the S1PR activation generates an S1P signalling pathway concerning vasoreactivity and vascular permeability, mediated by nitric oxide (NO), rendering a transient decrease of arterial pressure (Camm et al., 2014; Di Lorenzo et al., 2013). The vagomimetic effect consecutive to overstimulation of S1PR1 is limited due to β-arrestins activation, which induces receptor internalization and S1PR1 downregulation (Constantinescu et al., 2022b; Camm et al., 2014). S1PR desensitization generates the functional antagonist effect of S1PR modulators, which imposes a reduction of S1PR1 on the cell’s surface and changes S1P homeostatic signaling (Constantinescu et al., 2022b). These short-term effects of the S1PR modulator initiation were intensely studied and based mostly on fingolimod, the first oral immunomodulator from this class. Therefore, starting with siponimod, the titration dose was meant to induce the internalization (and downregulation) of S1PR at low doses, known to have a reduced vagomimetic effect (Legangneux et al., 2013).

Until now, there has been only one study on the long-term effects of siponimod on cardiac autonomic modulation. Twenty-six pwSPMS treated with siponimod for 6 months displayed an increase in sympathetic modulation of cardiac activity (Habek et al., 2022). Autonomic tests (e.g., Valsalva manoeuvre, deep breathing test, tilt test) were performed, and the time and frequency domain parameters of HRV (e.g., RMSSD, pNN50, SDNN, LF and HF) displayed reduced values after 6 months (Habek et al., 2022). After 6 months, the Valsalva ratio and respiratory sinus arrhythmia had lower values compared to initial evaluation, at M0. Therefore, the cardiovagal index, representing the beat-to-beat measurement of HR in response to sinusoidal deep breathing and the Valsalva maneuver, was significantly higher at M6 compared to M0. The autonomic regulation of BRS was not reported. These results on a 6 months follow-up underline that siponimod treatment, although it has a vagomimetic initial effect, in the long term, determines a shift towards sympathetic predominance in the autonomic cardiac modulation. Our results from a larger cohort of 47 pwSPMS showed a consistent shift in cardiac autonomic modulation under continuous treatment with siponimod over a 12-month follow-up period, and confirm the previous observations. This is the first analysis of findings on the long-term effects of siponimod treatment on cardiac autonomic modulation.

There is limited data available regarding the long-term effects of S1PR modulators on the sympathovagal balance. Most studies were conducted on fingolimod, which highlights the need for extended follow-up studies on second-generation S1PR modulators.

Some authors have described a persistent change in HRV lasting several months under treatment with fingolimod, thus emphasizing a continued parasympathetic predominance on the cardiac autonomic modulation (Akbulak et al., 2018). However, other results have proven the opposite. A shift towards a predominantly sympathetic HR modulation after 3 months of fingolimod treatment was described by Simula et al. (2016) A significant decrease in the BRS 6 months after fingolimod initiation and impairment of the parasympathetic and sympathetic cardiac control were also noted in the study of Racca et al. (2016) HRV parameters specific to the parasympathetic modulation of the HR, such as pNN50, RMSSD, and HF power, decreased significantly in this foregoing study, concomitantly with a decrease in LF power (Racca et al., 2016), probably due to an impairment of the central autonomic regulation and dysfunction of β-adrenergic receptor sensitivity.

Most studies have shown that S1PR modulators, particularly fingolimod, have an inverse short- and long-term effect on the ANS function. Hilz et al. have reported a significant increase in resting BRS that occurs within just one hour of starting fingolimod treatment (Hilz et al., 2017). It was discovered that even six hours after dosing, the values remained elevated. The highest BRS at rest was observed when fingolimod had lowered the HR and BP values to their nadir in response to the gradually increasing vagomimetic effects (Hilz et al., 2017). Similarly, siponimod initiation leads to an initial parasympathetic predominance, likely due to its well-known parasympathetic S1PR mediated effect (Habek et al., 2022). There is a contradictory effect between the increased autonomic activation during the early stages of drug use and the reduced autonomic activation during prolonged treatment with S1PR modulators (Camm et al., 2014).

Long-term treatment and chronic sympathetic activation can cause a decrease in HRV and reduced global cardiac autonomic modulation. Long-term use of S1PR modulators seems to affect BP control in pwMS, leading to a mild increase in BP (Camm et al., 2014). This suggests that prolonged treatment may lead to global cardiovascular autonomic dysregulation, independent of the acute response to the first dose of the drug.

Our findings indicate that over a long-term period of 12 months, there is a decrease in the parasympathetic modulation of the cardiac activity, starting from M1 and continuing towards M6. Simultaneously, there is an increase in sympathetic modulation in the first 6 months, followed by a tendency to recover towards the baseline values starting from M12. Various parameters reflect this change in the cardiac sympathovagal balance during the 12-month follow-up, mainly the time domain RMSSD and pNN50. The total power of HRV also showed a persistent and significant decrease starting from M1.

Moreover, in our study we assessed the cardiac autonomic modulation in response to specific autonomic challenge tests. The absence of tachycardia or slight increase in BP after a tilt test could suggest an inadequate sympathetic activation such as neurocardiogenic syncope, or orthostatic hypotension. The lack of bradycardia or a decrease in BP after deep breathing test or re-tilt test could indicate an impaired parasympathtic response. This could be the result of reduced BRS, or autonomic dysfunction rendering inability to adapt BP and HR to posture, orthostatic intolerance and overall cardiovascular instability. Reduced HRV often reflects an imbalance in the autonomic nervous system, particularly a dominance of the sympathetic over the parasympathetic nervous system. Reduced HRV is associated with various adverse cardiovascular outcomes, including increased risks of arrhythmias, heart failure, and mortality. It reflects the heart’s reduced ability to adapt to changing physiological demands, leading to a higher susceptibility to cardiovascular events and complications.

Our results indicate that during all evaluations performed in 1-year follow-up, pwMS under siponimod treatment presented normal cardiac responses to autonomic activation tests (tilt test and deep breathing test), explaining why there is no clinical phenotype of syncope or orthostatic hypotension in our cohort. This observation is sustained by the dynamics of the HRV and the hemodynamic parameters during the autonomic challenge. The tilt test determined a sympathetic activation, whereas the deep-breathing test imposed an increased vagal modulation of the HR. The time domain parameters RMSSD and pNN50 had eloquent variations of their values during the autonomic tests sustaining this observation. The frequency domain parameters are also in line with this finding. Deep metronomic breathing at a respiratory rate of 6 cycles/min is the most common means of eliciting parasympathetic activation. At this respiratory rate, the processing of the RRI signal shows a significant shift from the HF range to the LF range, as previously demonstrated (Malik, 1998a; Malik, 1998b). Interestingly, this shift from HF to LF (also reflected by an increase of LF/HF ratio) could be described for RRI similarly in all evaluation tests during the 12-month follow up. This result is in line with data from other studies which showed that voluntary control of breathing does not alter vagal modulation of HR (Patwardhan et al., 1995). The HF component as a marker of vagal modulation is respiration-mediated and thus determined by the frequency of breathing which was the same in all the autonomic tests in all the patients in our study. The modulation of BRS during tilt test was also present in all investigated pwMS. Orthostatic stress usually leads to a significant decrease in BRS, as previously described (Ducla-Soares et al., 2007). All the patients presented a physiological variation of the BRS during the autonomic tests. After the first month, there is a significant overall decrease in the BRS, with a slight recovery to M12. This finding can be further studied with the upcoming data in patients with siponimod treatment.

Hemodynamic and vascular parameters Cwk, TPR or ZaO illustrated normal variations during autonomic tests. Still, during the long-term follow-up, these parameters described a slight decrease in the intrinsic elastic properties of the arteries. It is known that the activity of the sympathetic branch of the autonomic nervous system is positively associated with peripheral resistance (Barnes et al., 2014). This observation should be further analysed to determine if it reflects physiological ageing or is a direct consequence of siponimod long-term treatment.

In parallel, few studies described the autonomic function using specific activation tests (e.g., deep breathing or tilt test) in long-term follow-up in pwMS, appart from treatment with S1PR modulators. According to their observation, there is a progression of autonomic dysfunction in MS over a 1-year follow-up, and this could correlate with progression in clinical disability, reflected in higher EDSS scores (Nasseri et al., 1998; Nasseri et al., 1999; Flachenecker et al., 2001; Habek et al., 2019). In one study both RRMS and SPMS patients were included (Nasseri et al., 1998). Moreover, magnetic resonance imaging (MRI) parameters seem to predict autonomic dysfunction in people with clinically isolated syndrome (Huwiler and Zangemeister-Wittke, 2018). Flachenecker et al. suggested that parasympathetic dysfunction is closely related to the progression of disability in pwMS, while sympathetic cardiac modulation impairment is associated with the clinical activity of the disease (Flachenecker et al., 2001). Based on this observation, it would be expected that sympathetic activation increases in people who experience relapses. In our study, pwSPMS were relapse-free during the autonomic evaluations. Therefore, the increase in sympathetic cardiac modulation, constantly seen from M1 to M6, found in our research in pwSPMS, regardless of relapses, could directly correlate to the impact of the siponimod treatment, and reflect the adjusting S1PR profile in cardiac myocytes in response to continuous treatment.

The parasympathetic effect is expressed before the internalization and desensitization of the S1PR1 by the S1PR agonist. Siponimod is also a selective functional antagonist of the S1PR1 subtype on the long run. This effect on S1PR1 is distinctive and not seen with the endogenous ligand S1P, which correspondingly internalizes S1PR1 upon binding but afterwards dissociates in endosomes (Constantinescu et al., 2022b). At the same time, the S1PR1 recycles back to the plasma membrane (Huwiler and Zangemeister-Wittke, 2018). After long-term S1PR1 modulation, consecutively to the functional antagonism, there is a shift towards S1PR2 and S1PR3 subtype activation (Camm et al., 2014), which can lead to vasoconstriction. A mild increase in BP after approximately 6 months of treatment (3 mmHg for SBP, 1.2 mmHg for DBP), staying stable after that, was described in siponimod-treated persons (European Medicines Agency, 2020). The agonistic effect on S1PR5, the other specific target receptor exerted by siponimod, is responsible for additional biological effects with less well-known outcomes in the long term (Huwiler and Zangemeister-Wittke, 2018).

Another aspect that should be considered is that pwMS exhibit similar age-related changes in autonomic cardiovascular regulation as healthy individuals, including an increase in sympathetic activity and a decrease in parasympathetic cardiac activity (Fluckiger et al., 1999; Brown et al., 2003).

Initially, sympathetic hyperactivity is a mechanism that compensates for the aging process. However, this chronic stimulation eventually becomes harmful to the cardiovascular system and β-adrenergic receptors, resulting in dysfunction in their signaling (de Lucia et al., 2018). The desensitization of β-adrenoreceptors caused by the phosphorylation of receptors followed by internalization and changes in G protein and kinase activity is associated with aging (Chadda et al., 2018). In cardiomyocytes, there is a reciprocal downregulation between β1-adrenoreceptors and the cardioprotective S1PR1 (Cannavo et al., 2017). The expression of S1PR2 is increased in endothelial senescence, while young endothelial cells express low levels of S1PR2. In contrast to S1PR2, S1PR1 has vasculoprotective effects (Trayssac et al., 2018).

The second generation of S1PR modulators, more selective regarding the S1PR activation, has fewer cardiovascular side effects at the initiation of the treatment. The dose titration proposed for siponimod and other second-generation S1PR modulators mitigates the impact on autonomic cardiac modulation, and these effects were well described in the literature (Constantinescu et al., 2022b). After long-term treatment with S1PR modulators, a decrease in the BRS, the capability to regulate sinus node activity, and a global impairment in cardiac autonomic control have been reported (Racca et al., 2019; Kappos et al., 2018). Again, most data come from fingolimod studies (Racca et al., 2016; Vehoff et al., 2017; Simula et al., 2015).

Our research underlines that siponimod, in the long run, has similar effects as fingolimod, determining a shift of the sympathovagal balance towards a more pronounced sympathetic cardiac modulation. This effect was also described in the study by Habeck et al. regarding the siponimod effect after 6 months of treatment (Habek et al., 2022). Our results emphasize that after 6 months of treatment, there is a sympathetic predominance in the cardiac modulation that tends to stabilize after 6 months towards 1-year follow-up. The continuation of our study for the next years following ANS responses of pwSPMS continuing or discontinuing siponimod will decide if the more extended treatment period, the autonomic sympathovagal balance will recuperate to the initial features before siponimod initiation.

The shift in cardiac autonomic modulation revealed by the HRV parameters in our study is most likely a pharmacological effect and not related to central autonomic disruptions in the context of MS, as patients did not encounter relapses during follow-up, and all patients remained clinically stable concerning the EDSS and did not have relevant MRI changes. Yet, an unspecific effect of other drug interactions for the disclosed changes in HRV cannot be excluded. Further studies with longer follow-ups should investigate whether sympathetic activation is maintained for more than 12 months or if the sympathovagal balance recovers to the pretreatment baseline. Another aspect that should be clarified wheather these long-term changes in the cardiac autonomic modulation are a consequence of the disease itself or represent effects of siponimod treatment.

The main limitations of this study are the small number of participants and the lack of a control group, which could have strengthened our study results and helped us better differentiate whether the changes observed in the cardiac autonomic function are related to medication or the disease itself. Our study aimed to document the impact on autonomic cardiovascular regulation in patients undergoing siponimod treatment, using the start of the treatment as a reference point. Moreover, additional data regarding the overall ANS function, such as the gastrointestinal, urinary or sexual activity, was not considered in our study. However, it would have allowed a better understanding of the sympathovagal balance in pwMS.

## 6 Conclusion

The vagomimetic effects of siponimod initiation are mitigated by initial dose titration over several days. However, in particular cases, cardiac monitoring is considered for patients at risk of developing cardiac arrhythmias or conduction abnormalities.

Continuous siponimod dosing shifts the cardiac autonomic regulation towards sympathetic predominance after 6 months, with stabilisation after a 1-year follow-up. Therefore, if the short-term effects of siponimod on cardiac autonomic modulation are mitigated due to dose titration, the long-term impact on cardiac autonomic modulation seems similar to fingolimod. The autonomic activation tests showed normal cardiovascular responses during 1-year follow-up in pwSPMS, confirming the safety profile of siponimod. Additional research on autonomic function could help clarify whether the observed sympathetic activation is merely a compensatory response, enhanced by the intervention in the S1P signaling pathway, or an inherent feature of the disease.

## Data Availability

The original contributions presented in the study are included in the article/supplementary material, further inquiries can be directed to the corresponding author.
